# Gene Conversion Violates the Stepwise Mutation Model for Microsatellites in Y-Chromosomal Palindromic Repeats

**DOI:** 10.1002/humu.22542

**Published:** 2014-03-07

**Authors:** Patricia Balaresque, Turi E King, Emma J Parkin, Evelyne Heyer, Denise Carvalho-Silva, Thirsa Kraaijenbrink, Peter de Knijff, Chris Tyler-Smith, Mark A Jobling

**Affiliations:** 1 UMR5288 CNRS/UPS—AMIS—Université Paul SabatierToulouse, France; 2 Department of Genetics, University of LeicesterLeicester, UK; 3 Eco-Anthropologie et Ethnobiologie, UMR 7206 CNRS, MNHN, Univ Paris DiderotParis, F-75005, France; 4 Wellcome Trust Sanger Institute, Wellcome Trust Genome CampusHinxton, UK; 5 MGC-Department of Human and Clinical Genetics, Leiden University Medical CentreThe Netherlands; 6 European Molecular Biology Laboratory, European Bioinformatics Institute, Wellcome Trust Genome CampusHinxton, Cambridge CB10 1SD, UK

**Keywords:** Y chromosome, gene conversion, palindrome, microsatellite, stepwise mutation model, DYS385

## Abstract

The male-specific region of the human Y chromosome (MSY) contains eight large inverted repeats (palindromes), in which high-sequence similarity between repeat arms is maintained by gene conversion. These palindromes also harbor microsatellites, considered to evolve via a stepwise mutation model (SMM). Here, we ask whether gene conversion between palindrome microsatellites contributes to their mutational dynamics. First, we study the duplicated tetranucleotide microsatellite DYS385a,b lying in palindrome P4. We show, by comparing observed data with simulated data under a SMM within haplogroups, that observed heteroallelic combinations in which the modal repeat number difference between copies was large, can give rise to homoallelic combinations with zero-repeats difference, equivalent to many single-step mutations. These are unlikely to be generated under a strict SMM, suggesting the action of gene conversion. Second, we show that the intercopy repeat number difference for a large set of duplicated microsatellites in all palindromes in the MSY reference sequence is significantly reduced compared with that for nonpalindrome-duplicated microsatellites, suggesting that the former are characterized by unusual evolutionary dynamics. These observations indicate that gene conversion violates the SMM for microsatellites in palindromes, homogenizing copies within individual Y chromosomes, but increasing overall haplotype diversity among chromosomes within related groups.

## Introduction

The male-specific region of the human Y chromosome (MSY) contains eight large inverted repeats (IRs; “palindromes”), each with two repeat copies (“arms”) separated by single-copy spacers [Skaletsky et al., 2003]. The arms of each palindrome show 99.94%–99.997% similarity in alignable sequence, as a result of frequent gene conversion activity [Rozen et al., 2003], in which nonreciprocal exchange homogenizes variants that arise through the slow processes of nucleotide substitution and insertion/deletion. Comparisons of orthologous palindromes in human, chimpanzee, and gorilla [Rozen et al., 2003; Hallast et al., 2013] indicate that conversion has been acting as a conservative force, significantly reducing interspecific divergence in arms compared with spacers.

These remarkable sequences make up about a quarter of the human Y-chromosomal reference sequence euchromatin (5.7 Mb out of 24 Mb), and are home to 26/166 (16%) of studied Y-chromosomal microsatellites that have 3–6-bp repeat units [Ballantyne et al., 2010]. Under the “classical” stepwise mutation model (SMM) [Ohta and Kimura, 1973], microsatellite mutations arise by replication slippage, usually generating a mutant allele differing from the parental allele by a single repeat unit, and much more rarely by two or more repeats. Furthermore, single-copy microsatellites show a clear length dependency of mutation rate, in which very small alleles can become “frozen” in an almost immutable state, but larger alleles mutate more rapidly [Ballantyne et al., 2010; Sun et al., 2012]. However, within palindromes, gene conversion, as well as stepwise mutation, might play a role in microsatellite dynamics and diversity. Conversion between alleles of very different lengths could in principle produce apparent multistep mutations, and could thereby reduce the length dependency of mutation rates. The extent to which any such conversion processes violate the SMM would depend on the relative rates of gene conversion and stepwise mutation. An additional factor might be the heterology represented by a large allele length difference between microsatellite copies within a palindrome: would the mismatch represented by such heterology inhibit gene conversion, such that conversion events would be restricted to alleles of similar lengths?

Here, we investigate the relative roles of conversion and stepwise mutation in influencing the diversity of microsatellite alleles. Choosing a specific tractable duplicated microsatellite, DYS385, we first show that the distribution of its allele combinations within SNP-defined haplogroups cannot be explained by a classical SMM, and that gene conversion events are therefore likely to occur between highly heterologous alleles. We then extend these findings by asking how the conversion we infer has affected the difference in repeat number between paralogous microsatellite copies within all palindromes in the MSY reference sequence, and show that this difference is significantly reduced compared with nonpalindromic-duplicated microsatellites. Gene conversion therefore has a widespread effect on the dynamics and diversity of duplicated Y chromosome microsatellites contained within palindromes.

## Materials and Methods

### DNA Samples

DNA samples were from Himalayan and Central Asian collections of the authors, and obtained with appropriate informed consent as described [Parkin et al., 2006; Parkin et al., 2007; Segurel et al., 2008; Kraaijenbrink et al., 2009]. Full Himalayan microsatellite data [Parkin et al., 2006; Parkin et al., 2007] and partial Central Asian microsatellite data [Segurel et al., 2008] were described previously.

### Y-Chromosome Haplotyping and Phasing of DYS385

Binary markers were typed using the SNaPshot minisequencing protocol on an ABI3100 capillary electrophoresis apparatus (both Applied Biosystems Inc., Foster City, CA). Amplification primers and SNaPshot primers were based on ones published previously [Paracchini et al., 2002; Bosch et al., 2006], with additional primers based on published sequences [Y Chromosome Consortium, 2002].

Twenty Y-specific microsatellites (DYS19, DYS385a/b, DYS388, DYS389I, DYS389II, DYS390, DYS391, DYS392, DYS393, DYS426, DYS437, DYS438, DYS439, DYS447, DYS448, DYS460, YCAIIa/b, and Y-GATA-H4.1) were typed in a multiplex [Butler et al., 2002]. PCR products were resolved on an ABI3100 capillary electrophoresis apparatus, and analyzed using GeneMapper software (Applied Biosystems). Allele nomenclature (Supp. Table S1) was as described [Parkin et al., 2006], and is consistent with ISFG recommendations [Gusmão et al., 2006].

Copy-specific amplification of DYS385 was carried out in a two-step procedure, as follows. Two initial amplifications, specific for DYS385a and b, respectively, were done in 10 μl reactions, using unlabeled copy-specific primers as described [Kittler et al., 2003]. Secondary reactions using standard fluorescently labeled DYS385 primers [Butler et al., 2002] were then carried out using 1 μl of each of the initial PCR products as templates. Labeled products were resolved by capillary electrophoresis.

### TMRCA Estimation

Mean TMRCA (with 95% confidence intervals) for the O3e, R2, and R1a haplogroups was estimated using the program BATWING (Bayesian analysis of trees with internal node generation [Wilson and Balding, 1998]). We used 16 microsatellites from our multiplex (having excluded the study locus DYS385 and the other duplicated marker YCAII) under a model of constant population size. Individual priors for microsatellite mutation rates were based on published pedigree and father–son pairs data [Bianchi et al., 1998; Kayser et al., 2000; Dupuy et al., 2004; Kurihara et al., 2004; Ballard et al., 2005; Budowle et al., 2005; de Souza Goes et al., 2005; Turrina et al., 2006; Domingues et al., 2007; Hohoff et al., 2007; Lee et al., 2007; Decker et al., 2008], as described [Balaresque et al., 2010]. Previously unpublished priors are as follows: normal (0.00009,0.00002) for DYS426, gamma (6,3805) for DYS437, gamma (3,3841) for DYS438, gamma (5,1214) for DYS460, and gamma (7,1799) for Y-GATA-H4.1.

### Simulations of DYS385 Mutation

To test whether homoallelic DYS385 allele combinations are the result of gene conversion, we forward-simulated genealogies with mutation [King and Jobling, 2009a], from the most likely ancestral (modal) allele combination of each haplogroup. Published data describing a total of nine mutations of DYS385a and 22 mutations of DYS385b among 8244 Y chromosome transmissions [Kayser et al., 2000; Dupuy et al., 2004; Kurihara et al., 2004; Ballard et al., 2005; Budowle et al., 2005; de Souza Goes et al., 2005; Gusmão et al., 2005; Turrina et al., 2006; Domingues et al., 2007; Hohoff et al., 2007; Lee et al., 2007; Decker et al., 2008] were used to establish probabilities of different mutational changes. We note that the published mutation studies did not carry out copy-specific amplification of DYS385a and b, but followed the convention by taking the smaller amplicon to be DYS385a and the larger b; we believe that any effect of this on mutational probabilities is likely to be small. For DYS385a, p(no mutation) = 0.99891, p(+1 repeat) = 0.00085; p(−1 repeat) = 0.00012; p(+2 repeats) = 0.00012. For DYS385b, p(no mutation) = 0.99733; p(+1 repeat) = 0.00206; p(−1 repeat) = 0.00049; p(−3 repeats) = 0.00012. Each simulation considered 1,000,000 lineages descending from the ancestral combination, and applied the above mutational parameters for three different numbers of generations (the mean, and the upper and lower 95% confidence interval limits of TMRCA for each haplogroup; see Table 1).

**Table 1:** Characteristics of Studied Haplogroup Samples

Haplogroup	O3e	R1a	R2
Sample size	986	186	85
Modal DYS385a,b allele combination	13,18	11,14	13,18
Modal *δr*	5	3	5
DYS385 allele size ranges	a [11–19]	a [10–14]	a [11–14]
	b [12–23]	b [11–17]	b [13–21]
Observed DYS385a,b homoallelic combinations	[12,12]; [13,13]; [14,14]; [18,18]; [19,19]	[11,11]; [14,14]	[13,13]
TMRCA/generations (mean [2.5–97.5 CI])	243 [129–441]	204 [68–546]	236 [53–856]
Mean conversion rate/events per generation	0.021–8.1 × 10^−3^	No evidence	0.014–7.3 × 10^−3^

To assess the significance of differences in the observed and simulated distributions of homoallelic combinations, we used a randomization test. From the simulated data, we extracted at random a number of homoallelic combinations equal to those in the observed data. This was repeated 200 times and the significance of the distance between observed and randomized data assessed using as a Chi-square test.

### Comparison of palindrome and nonpalindrome microsatellites

A list of polymorphic-duplicated tri-, tetra-, and pentanucleotides was extracted from the literature (Supp. Table S2). Genome coordinates and published information on palindromes [Skaletsky et al., 2003] were used to assign a proportion of these to palindromes, and the remainder assumed to lie in nonpalindromic repeats. The number of repeats in the reference sequence (and hence the number of repeat unit differences between copies *δr*) was extracted via the UCSC Genome Browser. The significance of the difference between classes of the proportion of microsatellites where *δr* = 0 was assessed using a Fisher exact test.

Dinucleotide microsatellites (Supp. Table S3) were ascertained by using the “Microsatellites” track within the UCSC Genome Browser, which includes cases with at least 15 uninterrupted repeats, and therefore highly likely to be polymorphic. Coordinates of palindromes and large IRs in which conversion is not evident were used to find appropriate duplicated microsatellites. Additional nonpalindrome-duplicated microsatellites were also investigated by using the “DGV Struct Var” and “Segmental Dups” tracks in the UCSC Genome Browser to find candidate-duplicated regions, and then using BLAST searches based on microsatellites within these regions to find additional Y-chromosomal copies. Comparison of the palindrome and nonpalindrome classes was carried out as described above.

Most of the Y chromosome reference sequence derives from a hg R1b chromosome [Skaletsky et al., 2003], but a 1,082-kb segment (chrY:14,288,568-15,370,557) derives from a hg G chromosome [Sun et al., 1999]; all our microsatellite comparisons involved the hg R1b majority only.

## Results

### Demonstrating Microsatellite Gene Conversion Within Palindrome P4

**Figure 1:**
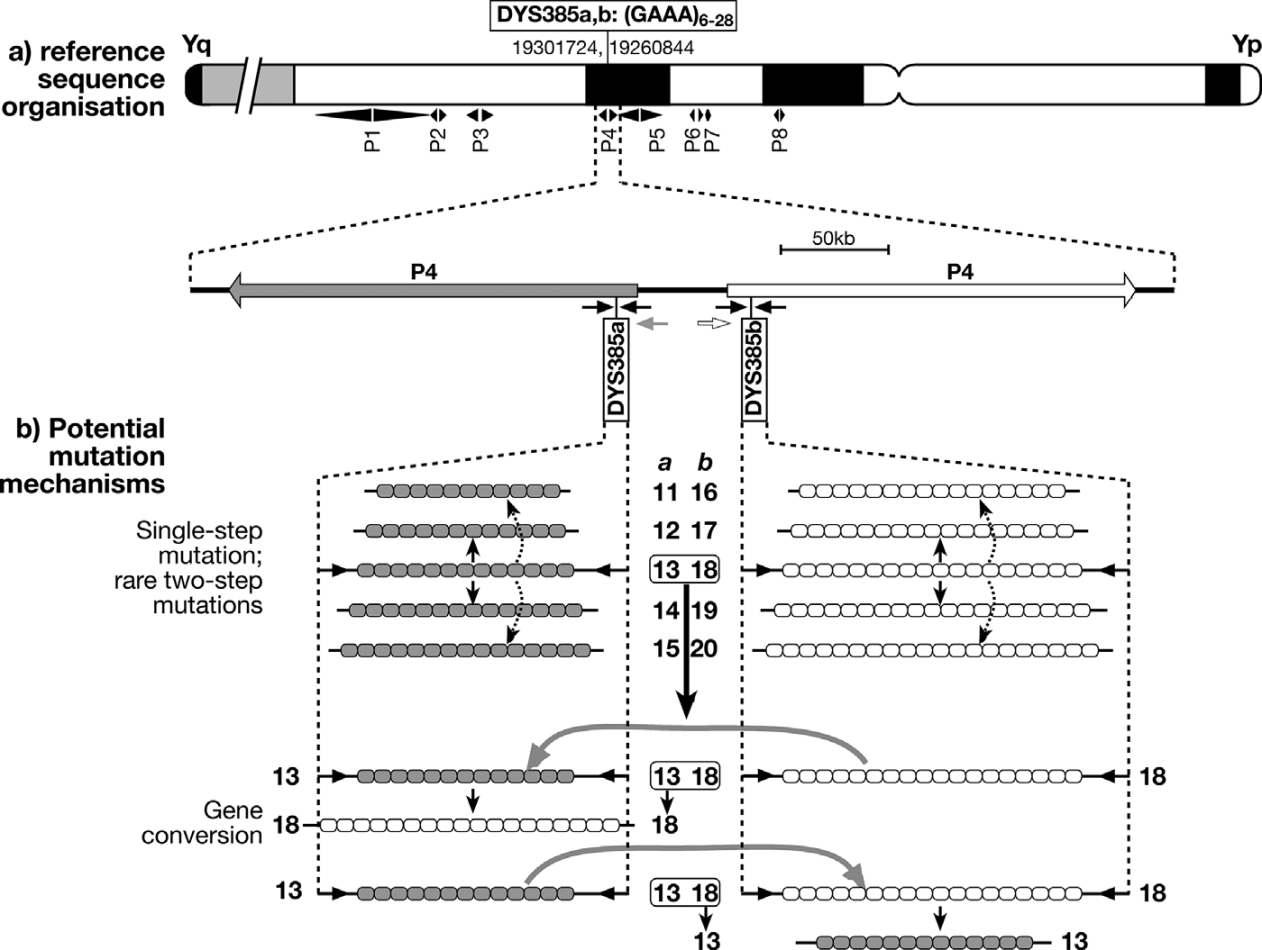
Genomic context of DYS385, and potential mutation processes. A: Reference sequence organization around DYS385, showing positions of the two copies of the microsatellite on an idiogram of the Y chromosome (with genome positions of start of each copy given according to build 36.1 of the reference assembly), and below, a schematic view of the region around DYS385. Large arrows indicate arms of the P4 palindrome (Skaletsky et al., 2003). Small arrows below indicate positions of PCR primers (not to scale) for standard amplification of both copies (black), or copy-specific amplification of DYS385a (gray) or b (white) (Kittler et al., 2003). B: Stepwise mutation via replication slippage normally gives +1 or −1 repeat products, and less frequently two-step (or greater—not shown) mutations. Gene conversion can lead to effective multistep mutations, in this example five steps.

As a study system to investigate the role of gene conversion, we chose the tetranucleotide repeat locus DYS385, which is included in forensic kits such as Y-filer (Applied Biosystems), and has been extensively used in population genetics and pedigree analyses, providing useful information about mutational behavior. This microsatellite exists in two copies in the Y chromosome reference sequence, the inner end of its repeat arrays lying 650 bp from the arm-spacer boundaries of palindrome P4 on Yq. By convention, the distal copy is known as DYS385a, and the proximal copy as DYS385b (Fig.1A). Although it is possible to amplify and type each copy independently using primers in the spacer region [Kittler et al., 2003], most of the large body of published data on this widely typed marker lacks this copy-specific information, and simply considers the smaller allele as DYS385a, and the larger as DYS385b. The total allele length range seen in DYS385a and b is 6–28 repeats (http://www.yhrd.org). We refer to a particular DYS385a,b state as an “allele combination,” to avoid confusion with “haplotype,” which we here reserve for single-copy microsatellites.

If gene conversion is acting upon DYS385, we expect it to convert heteroallelic combinations with large (e.g., >2 repeat) length differences between DYS385a and b, into homoallelic combinations. For example, the heteroallelic combination a,b = 13,18 might generate homoallelic 13,13 or 18,18 in a single conversion event within one generation, rather than via several stepwise events over multiple generations (Fig.1B). We hypothesized that, if this occurs, it might be discernible in the distribution of combinations within a lineage of related chromosomes that descend from a 13,18 common ancestor; conversion should generate extreme allele combinations (e.g., 13,13 and 18,18), possibly in the absence of intermediates (e.g., 13,15). To find suitable lineages for this test, we surveyed DYS385 allele combinations in a large set of Y chromosomes from our collections (data not shown) classified into the branches of the Y phylogeny based on binary markers (haplogroups). This survey identified three suitably well-resolved haplogroups in which the modal difference in repeats between DYS385a and b (*δr*) was >2 repeats (potentially large enough to distinguish conversion events from stepwise mutations), and for which we had sufficient numbers of chromosomes and also additional data for single-copy Y-microsatellites that would allow us to estimate the ages of the lineages (Supp. Table S1). These haplogroups (Table 1) were R1a (*δr* = 3), O3e (*δr* = 5), and R2 (*δr* = 5).

Despite their large modal *δr* values, each of these haplogroups contains examples of DYS385a,b homoallelic combinations, observed as a single, rather than a double, peak in an electropherogram. To exclude the possibility that these actually represent deletions of one of the DYS385 copies (hemiallelic), rather than the presence of two copies having the same repeat number, in each case we verified that both DYS385a and b could be amplified separately by PCR [Kittler et al., 2003].

Having identified these haplogroups and associated datasets, we asked whether the distribution of DYS385a,b combinations within each haplogroup fits the expectations from the SMM. First, we used the dating method BATWING [Wilson and Balding, 1998] to estimate the TMRCA (time to most recent common ancestor) of each set of chromosomes in the three haplogroups, using 16-locus haplotypes based on single-copy microsatellites, excluding DYS385. Next, we simulated mutation under a SMM (see *Materials and Methods*) starting from a most parsimonious ancestral DYS385a,b allele combination for each haplogroup that was equivalent to the modal combination, and considering the mean value and the upper and lower 95% confidence limits of the TMRCA, to give three simulated distributions of allele combinations (and *δr* values) in each case.

**Figure 2:**
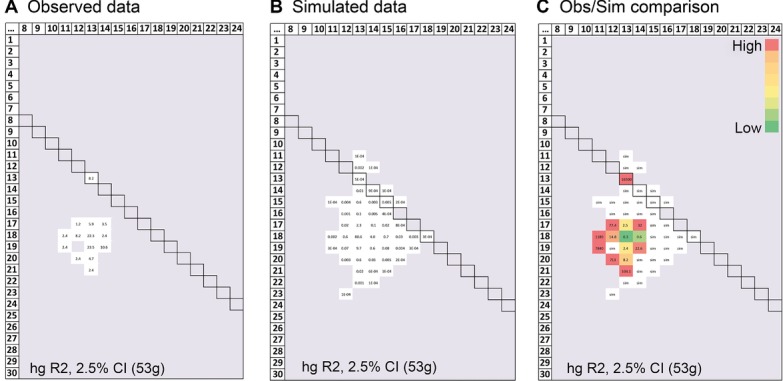
Comparing DYS385a,b allele combinations in observed and simulated data using heat-maps. For the example of haplogroup R2 and a TMRCA of the lower confidence interval limit (2.5% CI) of the BATWING estimate (53 generations), (A) observed, and (B) simulated DYS385 combinations are displayed in a matrix with DYS385a on the y-axis and b on the x-axis. The boxed diagonal highlights homoallelic combinations, and numbers (%) in cells indicate frequency. C: The observed and simulated data merged. “sim” in a white cell means this combination is only found in the simulated data, never in the observed data. Numbers indicate the ratio of frequencies in observed compared with simulated data. Colors emphasize the range of values (green for the lowest, and red for the highest).

**Figure 3:**
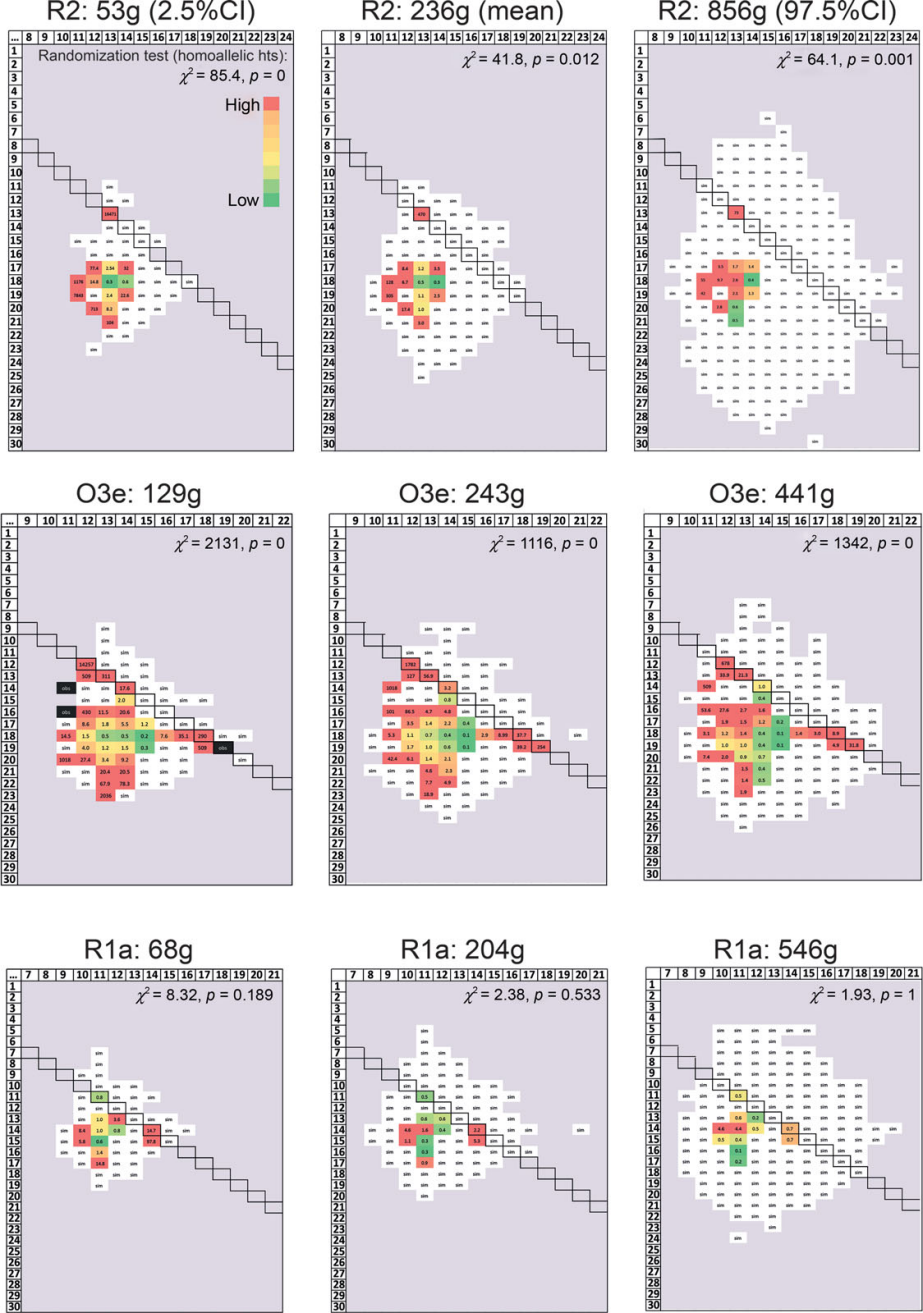
Heat-maps for observed and simulated DYS385a,b allele combinations. Data are displayed as in Figure2C, for three haplogroups and three TMRCA estimates. “sim” in white cell: combination only found in simulated data, never in observed data. “obs” in black cell: combination only found in observed data, never in simulated data. Colors and numbers within cells are as in Figure2.

To compare the observed and simulated data, we display them as heat maps. Figure2 shows an example for hg R2 for one value of the TMRCA, in which observed and simulated data are shown separately (Fig.2A and B), and then merged (Fig.2C). This latter representation is presented in Figure3 for all three haplogroups, each at three TMRCA values. Although homoallelic combinations are generated in the simulations, their allele length distributions differ from those that are observed. For example, in the observed data for hg O3e, the homoallelic combinations are seen for alleles 12, 13, 14, 18, and 19, but not for the intermediate alleles 15, 16, and 17; in the simulated data, the intermediates always appear. Using a randomization test, the observed distributions of homoallelic combinations are significantly different from the simulated distributions for all TMRCAs for haplogroups R2 and O3e (Fig.3). For hg R1a, the difference is never significant, which reflects either a lack of gene conversion acting on sampled chromosomes within this haplogroup, or more probably the effect of a relatively low modal *δr* of 3, compromising our ability to distinguish conversion events from stepwise processes.

### Multiple Gene Conversion Events Within Haplogroups, and Gene Conversion Rate Estimates

Do the homoallelic combinations reflect single or multiple ancestral conversion events? Clearly, when homoallelic combinations carrying both long and short alleles exist within a haplogroup, and where *δr* is large, this indicates at least two events. An example is hg O3e, which has a modal combination of 13,18, but contains both 13,13 and 18,18 combinations. However, the 13,13 cases, for example, could either all be identical by descent, reflecting a single ancestral conversion event, or alternatively could have arisen via separate events.

**Figure 4:**
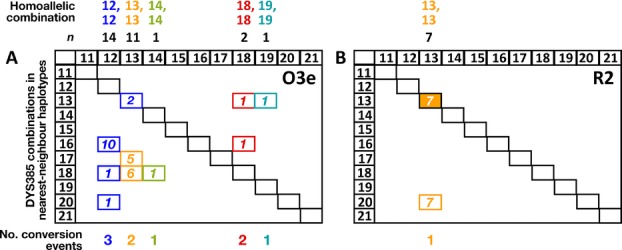
Nearest neighbors of haplotypes carrying DYS385a,b homoallelic combinations tend to carry heteroallelic combinations. Homoallelic combinations are indicated at the top of each panel for (A) hg O3e, and (B) hg R2, with the numbers of chromosomes (*n*) shown. In the squares below are indicated the DYS385 allele combinations carried by the nearest haplotypic neighbors based on 16-locus single-copy microsatellite haplotypes. Colors correspond to the homoallelic combinations, and numbers in small boxes indicate numbers of chromosomes. In most cases, the nearest neighbors of homoallelic cases are heteroallelic for DYS385 (e.g., there are 10 instances of 12,16 combinations) and differ from two up to eight repeats; this indicates that conversion, rather than single-step mutation, is most likely responsible for the homoallelic cases. At the bottom is given the minimum number of gene conversion events for each homoallelic combination.

To address this, for each Y chromosome characterized by a homoallelic combination for DYS385a,b, we identified the most similar chromosomes within the same haplogroup using its 16-locus single-copy microsatellite haplotype. Then, we examined the DYS385a,b combinations carried by these nearest neighbors. Are they similar to one another, suggesting a single gene conversion event, or diverse, suggesting multiple events? Figure4A shows, for hg O3e, the DYS385a,b combinations carried by the chromosomes having the nearest neighbor 16-locus haplotypes. For example, the 14 haplotypes displaying a 12,12 combination are most closely related to four different haplotypes, carrying 12,16, 12,18, and 12,20 and 13,13 combinations. The most parsimonious scenario suggests three probable conversion events, 12,16 to 12,12 (−4 repeats), 12,18 to 12,12 (−6 repeats), and 12,20 to 12,12 (−8 repeats), respectively. The equally closely related haplotype carrying the combination 13,13 could be due to conversion, but may also have arisen from 12,12 by stepwise mutation, and to be conservative is excluded. This approach allows us to identify nine probable conversion events for hg O3e, and one event for hg R2 (Fig.4B).

We also wished to compare the average DYS385 gene conversion rate with the single-step mutation rate. The conversion rate cannot be determined with any precision, because of uncertainty over the number of generations encompassed by the studied sets of chromosomes, but the range in which the true value must lie can be estimated. For example, for hg O3e 986, Y chromosomes were studied (Table 1). The smallest possible number of encompassed generations is 1,114, in which all the sampled males are brothers, and the minimum haplogroup TMRCA estimate (129 generations) is considered ([1*986]+[129−1]). Conversely, the largest number is 434,826, in which all sampled males descend independently from the MRCA, and the maximum TMRCA estimate (441 generations) is considered (968*441). For hg O3e, the observed nine events then equate to an average gene conversion rate range of 0.021–8.1 × 10^−3^ per generation. A similar procedure for hg R2 yields a range of 0.014–7.3 × 10^−3^ per generation. It seems more likely that the true conversion rate for DYS385 must be at the lower end of this range, and markedly lower than the single-step mutation rate, otherwise a high *δr* could not be maintained.

### Distinct Mutational Dynamics at DYS385 Compared with Single-Copy Loci

The above analysis indicates that the observed homoallelic combinations for alleles 12, 13, 14, 18, and 19 of DYS385a,b are more likely to be generated by gene conversion than by a SMM process. Is the impact of conversion on DYS385 also evident in comparisons with single-copy microsatellites?

**Figure 5:**
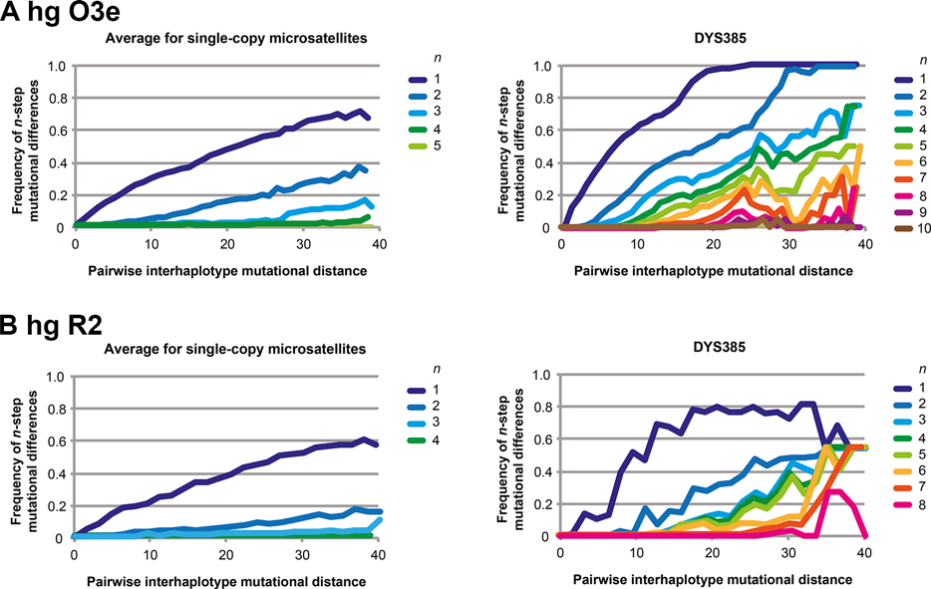
The frequency of *n*-step mutational differences in pairwise haplotype comparisons for different classes of microsatellites. For (A) hg O3e and (B) hg R2, graphs show the frequency of mutational differences in pairwise interhaplotype comparisons of increasing numbers of mutational steps. Probabilities are shown for single-copy microsatellites (averaged across loci), and the palindromic duplicated microsatellite DYS385. The latter shows an excess of multistep mutations, reflecting likely gene conversion activity.

To address this, we compared all haplotypes pairwise for hgs O3e and R2, respectively, using the 16 single-copy microsatellites, and categorizing by the number of mutational steps (from 0 to 40) between haplotypes. For each interhaplotype distance class, and for each microsatellite, we then considered the frequency of mutational differences (e.g., 1-step, 2-step, and so on). Figure5 shows the results of this comparison for each haplogroup—we plot separately the results for single-copy microsatellites, averaged across loci (left panels), and for the palindromic-duplicated microsatellite DYS385 (right panels). For both haplogroups, the single-copy loci average no more than four mutational steps difference, no matter how great the interhaplotype distance. However, DYS385 shows up to 10 mutational steps difference, which is consistent with large apparent mutations arising through gene conversion.

From this analysis, we conclude that the mutational dynamics of the DYS385a,b palindromic-duplicated microsatellite differs from that of single-copy loci; this difference is likely to reflect the impact of gene conversion, a process that is particularly active in Y-chromosomal palindrome structures.

### General Influence of Gene Conversion on Microsatellite Diversity Within Y Palindromes

Having demonstrated that gene conversion influences the diversity and dynamics of a specific palindromic microsatellite, DYS385, we can ask whether there is evidence for a more general influence of conversion on the many microsatellites that lie within MSY palindromes. In the case of DYS385, we sought lineages in which *δr* was large, but we hypothesized that these cases should be atypical, in that gene conversion would actually tend to reduce the *δr* for palindromic microsatellites by repeatedly returning heteroallelic combinations to the homoallelic state. To test this idea, we compared a set of palindromic microsatellites with a set of duplicated microsatellites that do not lie within Y-chromosomal palindromes. Note that this can only be done in the single Y chromosome comprising the reference sequence, since suitable population microsatellite and haplogroup data for duplicated microsatellites other than DYS385 are not currently available. Furthermore, the reference sequence has the advantage of allowing us to distinguish unambiguously between homoallelic (e.g., 14,14) and hemiallelic (e.g., 14,null) combinations.

First, we surveyed the list of duplicated polymorphic tri-, tetra-, and pentanucleotide microsatellites that have been exploited for Y chromosome diversity studies (Supp. Table S2). This yielded 25 palindrome-located microsatellites (in addition to DYS385), and six duplicated nonpalindrome microsatellites that encompass a total of 13 copies. Measurement of *δr* within the MSY reference sequence gives a mean value of 0.77 repeats for the palindromic examples (14/26 having *δr* = 0), and 2.15 repeats for the nonpalindromic cases (only one having *δr* = 0). Considering the proportions of *δr* = 0, this difference is significant despite the small sample size (Fisher exact test; odds ratio: 0.0759, *P* = 0.006**).

We further extended this analysis by considering duplicated dinucleotide microsatellites in the MSY reference sequence for which at least one copy had 15 uninterrupted repeats, and was therefore highly likely to be polymorphic (Supp. Table S3). A total of 51 such dinucleotide microsatellites in palindromes have mean *δr* of 1.4, and *δr* = 0 for 23/51 cases; the corresponding figures for the 28 nonpalindrome loci are mean *δr* of 6.4, and *δr* = 0 for 2/28. Again, considering the *δr* = 0 proportions, this difference is significant (Fisher exact test; odds ratio: 0.0961, *P* = 0.00038).

## Discussion

Our analysis of the diversity of allele combinations of the duplicated microsatellite DYS385, lying within palindrome P4, shows that gene conversion between palindrome arms, as well as “classical” SMM processes, plays an important role in its mutational dynamics. Furthermore, the comparison of other palindromic-duplicated microsatellites with duplicated MSY microsatellites that do not lie in palindromes, and which are therefore unlikely to be involved in frequent conversion, indicates that gene conversion has a pervasive effect on the mutational dynamics of these loci.

As with nontandemly repeated DNA, there is a paradox in that the more rapid gene conversion is, the more difficult it becomes to detect conversion events involving duplicated microsatellites, because the process will tend to homogenize the two copies. For DYS385, the strength of evidence for gene conversion depends on which haplogroup is surveyed, because each has a characteristic value of *δr*, and smaller *δr* values make conversion more difficult to distinguish from stepwise processes. The evidence is strongest for the two studied haplogroups with *δr* = 5. In addition, our simulation approach ignores demographic history and sampling effects, which clearly could contribute to uncertainty about the underlying mutation processes. Nonetheless, conversion is convincingly contributing to DYS385a,b diversity, and heterology of five repeat units (equivalent to a 20-bp indel) difference clearly does not present an insuperable barrier to conversion.

Is there any independent evidence to support the idea of gene conversion at DYS385? In a published dataset [Malyarchuk et al., 2010] for haplogroup C3d (C-M407) 168/182, Y chromosomes carry the combinations 11,17, 11,18, and 11,19, but the remaining 14 closely related chromosomes carry the combination 11,11. The authors speculate about deletion of DYS385a or b, or mutation in a primer-binding site, but gene conversion seems a more likely explanation. Additional evidence could come from direct observations of conversions among mutation data in pedigrees or father–son pairs: the YHRD describes 54 mutation events, and include one case where the combination 12,14 gives rise to 14,14 in a single event—either a two-step mutation via slippage, or a conversion event [Budowle et al., 2005].

Can anything be said about the relative rates of gene conversion and SMM processes at DYS385? Clearly, individual conversion events must be less common than single-step mutations, otherwise a high *δr* could not be maintained. This is supported by inspection of the published pedigree mutation data, although sometimes (depending on the paternal combination) single-step mutation and conversion would be indistinguishable. Our estimates of the likely rate range of gene conversion suggests a lower limit of ∼10^−5^ events per generation, some two orders of magnitude slower than single-step mutation. Notably, however, a single rare conversion event (e.g., from 11,18 to 11,11) could have a disproportionate impact on diversity because it would be equivalent to many single-step mutations.

Commercial kits for the typing of Y microsatellites were established for forensic purposes, yet have become increasingly applied in population genetic studies. So far, with the exception of DYS385, these have avoided multicopy microsatellites. However, the recent identification [Ballantyne et al., 2010] of 13 “rapidly mutating” loci (RM Y-STRs) and the likely development of kits based on these [Ballantyne et al., 2012], will introduce several highly variable multicopy microsatellites as possible population-genetic (and forensic) tools. Four RM Y-STRs are multicopy, and at least two of these, DYF387 and DYF399, lie in palindromes (Supp. Table S2). Commercial genealogical testing services type up to 111 Y-chromosomal microsatellites, including multicopy palindromic microsatellites to identify close patrilineal relatives, and to estimate time to most recent common ancestor between them [King and Jobling, 2009b]. These multicopy loci are potential targets for gene conversion, and likely to break the rules of conventional mutational models, introducing bias into measures of interhaplotype distances. The temptation to use them simplistically in population studies should therefore be resisted; characterization of gene conversion acting on these loci will be worthwhile, and allow them be integrated into microsatellite evolutionary models.

Genetic distance measures for haplotypes that include duplicated microsatellites are influenced by the fact that these loci are not normally typed in a copy-specific manner [Balaresque et al., 2007]. In addition, such measures calculated using single-copy microsatellites, and those including duplicated palindromic microsatellites, are expected to be very different, thanks to the effects of gene conversion. The impact of gene conversion on the latter would be particularly important when statistical indices including molecular distances between haplotypes (such as *R*_ST_), are used. Coalescence measures would also be affected by gene conversion, with the inclusion of palindromic microsatellites affecting TMRCA. For both *R*_ST_ and TMRCA, the direction of any effect would depend on the composition of the sample and the nature of the converted haplotypes. Depending on the geographical area investigated (and hence the predominant haplogroups), the impact of gene conversion on genetic estimators could either be negligible (haplogroups with *δr* ≤ 3 such as R1a or R1b1b2), or marked (haplogroups with *δr* > 3 such as R2, O3e, or some sublineages of I). Because of this influence of haplogroup and modal allele combinations, the impact of gene conversion is not simple [Balaresque et al., 2007]. Working in a well-defined phylogenetic context is important to gain a clear picture of the effects of gene conversion processes.

**Figure 6:**
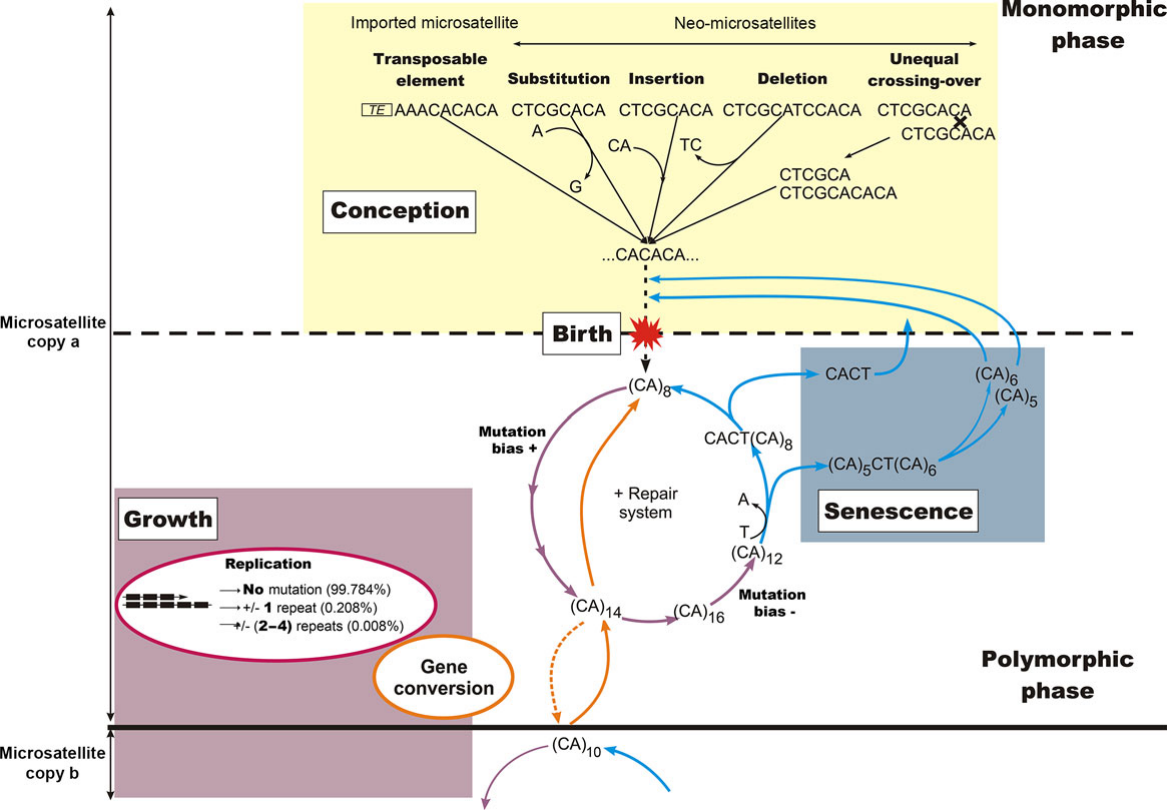
Incorporation of gene conversion into the classical life cycle of microsatellite evolution. A microsatellite sequence passes through two phases: a monomorphic phase (top), in which the repeat copy number is too low to represent a potential substrate for polymerase slippage and change in length; a polymorphic phase, in which copy number is sufficient (≥8 repeats) and is subject to gain and loss of repeats (with a mutation bias toward gains for smaller repeat arrays, and toward losses for larger arrays). Interruption of the repeat array can return the microsatellite to a monomorphic phase. Gene conversion with another microsatellite copy (bottom) can cause the gain or loss of several repeats in a single event, facilitating the transition from one phase of the life cycle to the other.

Finally, the recognition of gene conversion as a force acting on microsatellite evolution adds an additional layer of complexity to the traditional picture of how this class of loci evolves. Microsatellites are truly dynamic sequences, since their molecular structures change over time, driven by multiple evolutionary processes. Both repeat unit length and repeat number directly influence their polymorphism and mutation rates (reviewed by [Schlotterer, 2000]). This dynamic nature has motivated the comparison [Buschiazzo and Gemmell, 2006; Balaresque, 2007] of microsatellite evolution with a life cycle (Fig.6). Below a repeat number threshold [Messier et al., 1996; Rose and Falush, 1998], which varies across species [Balaresque et al., 2003] but lies close to seven or eight uninterrupted repeats, a microsatellite sequence is not considered to be polymorphic. When the microsatellite repeat number reaches its maturity threshold, it begins its life as polymorphic sequence. The microsatellite is then in a growth phase gaining and losing one, and occasionally, two repeats, but with an overall bias to expansion. It will stay “alive” as long as a minimal block of eight repeats remains uninterrupted by point mutation (which can send it back into a monomorphic state [Taylor et al., 1999]). By adding or removing up to eight repeats at once, gene conversion can be considered as an additional evolutionary force, with the power to instantly driving a microsatellite copy from a dead (monomorphic) to a live (polymorphic) phase of the life cycle, or vice versa. It remains to be determined whether such gene conversion includes any bias toward smaller or larger alleles, making it a net force for either the extinction or resuscitation of polymorphic microsatellites.

## Supporting Information

Disclaimer: Supplementary materials have been peer-reviewed but not copyedited.

Table S1. Y-chromosomal haplotype data is available as an Excel file under the Supporting Information for this article.

Table S2. Y-chromosome-wide non-dinucleotide microsatellites. Table S3. Y-chromosome-wide dinucleotide microsatellites.
