# Supplementary material for: Gene Conversion Violates the Stepwise Mutation Model for Microsatellites in Y-Chromosomal Palindromic Repeats
Source: Hum Mutat. 2014 Mar 7;35(5):609–17. doi: 10.1002/humu.22542 (PMC4233959; doi:10.1002/humu.22542)
Supplement: Supplementary file 2 [file humu0035-0609-SD2.pdf]

Supp. Table S2. Y-chromosome-wide non-dinucleotide microsatellites

No.	GBD ID	Chr. band location	Palindrome, Element, Strata	Localisation details (retroposon, gene…)	Copy-no. from Y-chr location (UCSC)	Repeat type	Repeat Structure (Kayser et al. 2004, Ballantyne et al. 2010)	Predicted amplicon size (UCSC) GRCh37/hg19 human	Ref. human allele	Amplicon size (UCSC) CGSC 2.1.3/panTro3 chimpanzee	Ref. chimp. allele	Ref.	Locus 1	Locus 2	δr
NON PALINDROMIC REPEATS (not necessarily symmetric)															
1	**DYF405S1**	Yq11.21/Yq11.2	other strata	Alu R	multi-copy	4	**(GGAA)**$_{4-14}$N$_{115}$(GGAA)$_3$(GAAA)$_1$(GGAA)$_3$	chrY:13983492-13983808 317bp chrY:7445801-7446093 293bp	11, 6	No matches because of mismatches in the primer binding sites and in the STR	-	Kayser et al. 2004, Ballantyne et al. 2010	11	6	5
2	**DYF403S1a**	Yp11.2/Yp11.1	IR3p, IR3d, IR3p, IR3d	Alu R	multi-copy	4	**(TTCT)**$_{10-17}$**N**$_{2-3}$**(TTCT)**$_{3-17}$	chrY:9,519,608+9,519,948 341bp chrY:6,225,842+6,226,153 312bp	17, 13	No matches	-	Kayser et al. 2004, Ballantyne et al. 2010; Ballantyne et al., 2011	17	13	3
3	**DYF403S1a**	Yp11.2/Yp11.1	IR3p, IR3d, IR3p, IR3d	Alu R	multi-copy	4	**(TTCT)**$_{10-17}$**N**$_{2-3}$**(TTCT)**$_{3-17}$	chrY:9,654,225-9,654,540 316bp chrY:6,347,622-6,348,058 437bp	16, 13	No matches	-	Kayser et al. 2004, Ballantyne et al. 2010; Ballantyne et al., 2011	16	13	3
4	**DYF384S1**	Yp11.2/Yq11.223	other strata	LTR25	multi-copy	3	**(TTG)**$_{8**}$N$_7$(GTTT)$_{4**}$	chrY:8973697-8973829 133bp chrY:23889928-23890057 130bp	8, 7	No matches	-	Kayser et al. 2004	8	7	1
5	**DYF399S1**	Yq11.23	P1d, (between P3-P2), P1p	MER61 (LTR) (TTTY4 non-coding gene)	multi-copy	4	(GAAA)$_3$N$_{7-8}$**(GAAA)**$_{10-23}$	chrY:26730126-26730414 289bp chrY:25096369-25096670 302bp chrY:27231970-27232262 293bp	17,18, 20	No matches because of mismatches in the primer binding sites and of a big gap due to a length difference in the STR	-	Kayser et al. 2004, Ballantyne et al. 2010; Ballantyne et al., 2011	multipl e	multipl e	(17/20=3), (17/18=1), (18/20=2) = 2
6	**DYF386S1**	Yq11.223/Yq11.2 3	P1, P3	Alu L	multi-copy	3	**(AAT)**$_{7-16}$	chrY:25777798-25777922 125bp chrY:24168505-24168620 116bp chrY:24710061-24710179 119bp chrY:28184450-28184574 125bp	14, 11, 12, 14	chrY:11514084+1151417 9 96bp chrY:3615591+3615707 117bp chrY:10671280-10671393 114bp chrY:2702522-2702638 117bp	7 14 13 14	Kayser et al. 2004, Ballantyne et al. 2010	multipl e	multipl e	(14/11=3), (14/12=2), (14/14=0), (11/12=1), (11/14=2), (12/14=2) =1.7 (2)

13 events

Mean δr =2.15

No.	GBD ID	Chr. band location	Palindrome, Element, Strata	Localisation details (retroposon, gene…)	Copy-no. from Y-chr location (UCSC)	Repeat type	Repeat Structure (Kayser et al. 2004, Ballantyne et al. 2010)	Predicted amplicon size (UCSC) GRCh37/hg19 human	Ref. human allele	Amplicon size (UCSC) CGSC 2.1.3/panTro3 chimpanzee	Ref. chimp. allele	Ref.	Locus 1	Locus 2	δr
PALINDROMIC REPEATS (symmetric)															
1	**DYF383S1**	Yq11.223/Yq11.2 3	P1	Alu L and R	multi-copy	3	(TCA)$_2$(TAG)$_1$**(TAA)**$_{9**}$	chrY:25889785+25889928 144bp chrY:28072450-28072593 144bp	9,9			Kayser et al. 2004	9	9	0
2	**DYF387S1**	Yq11.223/Yq11.2 3	P1	Alu L	multi-copy	4	(AAAG)$_3$(GTAG)$_1$(GAAG)$_4$N$_{16}$(GAAG)$_9$**(AAAG)**$_{13**}$	chrY:25931440+25931696 257bp chrY:28030679-28030939 261bp	13,13			Kayser et al. 2004, Ballantyne et al. 2010; Ballantyne et al., 2011	13	13	0
3	**DYF388S1**	Yq11.23/Yq11.22 3	P1	Alu R	multi-copy	4	(CTTC)$_6$**(CTTT)**$_{5-19}$N$_{18}$(CTTC)$_3$(TTTC)$_1$(CTTC)$_3$N$_8$(CTTC)3N$_{32}$(CTTC)$_3$	chrY:28076415+28076746 332bp chrY:25885624-25885963 340bp	13,15			Kayser et al. 2004, Ballantyne et al. 2010	13	15	2
4	**DYF390S1**	Yq11.23/Yq11.23	P1	LINE1 R (LTR L)	multi-copy	4	**(TTTA)**$_{9-14}$	chrY:26440453+26440671 219bp chrY:27521766-27521988 223bp	10,11			Kayser et al. 2004, Ballantyne et al. 2010	10	11	1
5	**DYF380S1**	Yq11.223/Yq11.2 3	P1	LTR ERV1 family L	multi-copy	3	**(AAT)**$_{8-11}$	chrY:25980114+25980302 189bp chrY:27982085-27982273 189bp	10,10			Kayser et al. 2004, Ballantyne et al. 2010	10	10	0
6	**DYF381S1**	Yq11.23	P1	LINE family L1	multi-copy	3	**(TTG)**$_{7-8}$	chrY:28101203+28101454 252bp chrY:25860914-25861165 252bp	8,8			Kayser et al. 2004, Ballantyne et al. 2010	8	8	0
7	**DYF385S1**	Yq11.223/Yq11.2 3	P1	TTTY3 Non coding genes	multi-copy	3	**(TTA)**$_{10}$	chrY:26084801+26085002 202bp chrY:27877404-27877608 205bp	10,11			Kayser et al. 2004	10	11	1

8	**DYF386S1**	Yq11.223/Yq11.23	P1	Alu L	multi-copy	3	**(AAT)$_{7-16}$**	chrY:25777798+25777922 125bp chrY:28184450-28184574 125bp	14,14			Kayser et al. 2004, Ballantyne et al. 2010	14	14	0
9	**DYF386S1(2)**	Yq11.223/Yq11.23	P3	Alu L	multi-copy	3	**(AAT)$_{7-16}$**	chrY:24168505+24168620 116bp chrY:24710061-24710179 119bp	11,12			Kayser et al. 2004, Ballantyne et al. 2010	11	12	1
10	**DYF395S1**	Yq11.221	P5	Alu L, LINE1 R	multi-copy	3	**(AAC)$_{13-14}$**	chrY:19739185+19739426 242bp chrY:20440348-20440589 242bp	13,13			Kayser et al. 2004	13	13	0
11	**DYF396S1**	Yq11.23	P1	LINE1	multi-copy	3	**(TCT)$_{6-9}$**	chrY:27727483+27727669 187bp chrY:26234761-26234947 187bp	8,8			Kayser et al. 2004, Ballantyne et al. 2010	8	8	0
12	**DYF397S1**	Yq11.23/Yq11.223	P1	Alu R	multi-copy	3	**(TAT)$_{14**}$**	chrY:28184380+28184574 195bp chrY:25777798-25777992 195bp	14,14			Kayser et al. 2004	14	14	0
13	**DYF397S1**	Yq11.23/Yq11.223	P3	Alu R	multi-copy	3	**(TAT)$_{14**}$**	chrY:24709991+24710179 189bp chrY:24168505-24168690 186bp	12,11			Kayser et al. 2004	12	11	1
14	**DYS527 a/b**	Yq11.223/	P1	Alu L	multi-copy	4	(GAAA)$_4$(AGAA)$_1$(GGAA)$_3$(ATGA)$_1$(AACA)$_1$(AGAA)$_1$(AGGA)$_1$(AAGA)$_{14}$**(AAGG)$_{28-38}$**(AAAG)	chrY:25885604+25885963 360bp chrY:28076415-28076766 352bp	7,7			Hanson et al. 2006; Hanson et al. 2007 (not in Kayser et al. 2004)	7	7	0
15	**DYF401S1**	Yq11.223	P1	Alu L	multi-copy	4	(AAGG)$_3$(AAGC)$_1$(AAGG)$_3$N$_{39}$(AAGG)$_3$N$_8$(AAGG)$_3$(AAAG)$_1$(AAGG)$_3$N$_{13}$**(AAAG)$_{8-23}$**G(AAGG)$_6$	chrY:25885604+25885963 360bp chrY:28076415-28076766 352bp	15,13			Kayser et al. 2004, Ballantyne et al. 2010	13	15	2
16	**DYF404S1**	Yq11.223	P1	Alu R	multi-copy	4	**(TTTC)$_{10-20}$**N$_{42}$(TTTC)$_3$	chrY:25954055+25954251 197bp chrY:28008132-28008320 189bp	16,14			Kayser et al. 2004, Ballantyne et al. 2010; Ballantyne et al. 2011	14	16	2
17	**DYF410S1**	Yq11.23	P1	LINE1 L, LTR ERVL family R	multi-copy	4	**(AAAT)$_{7-13}$**	chrY:27521766-27522040 275bp chrY:26440401-26440671 271bp	11,10			Kayser et al. 2004, Ballantyne et al. 2010	10	11	1
18	**DYS459 a/b**	Yq11.223 - Yq11.23	P1	Alu R	multi-copy	4	**(TTAT) <=(ATTT)$_{6-11}$**	chrY:26078790-26078941 152bp chrY:27883469-27883616 148bp	10,10			Redd et al. 2002; Hanson et al. 2006; Hanson et al. 2007; Ballantyne et al. 2010 (not in Kayser et al. 2004)	10	10	0
19	**DYS464 a/b/c/d**	Yq11.23	P2	Alu R	multi-copy	4	**(CCTT)$_{9-20}$**N$_{46}$(CCTT)$_3$N$_8$(CCTT)$_4$	chrY:27087569-27087835 267bp chrY:25471897-25472175 279bp chrY:26874567-26874841 275bp chrY:25240822-25241096 275bp	15,15,15,15			Redd et al. 2002; Hanson et al. 2006; Ballantyne et al. 2010 (not in Kayser et al. 2004)	15	15	0
20	**DYS385a/b**	Yq11.222	P4	bi-local / Alu L	multi-copy	4	**(AAGG)4N14(AAAG)3N12(AAAG)3N29(AAGG)6-7(GAAA)7-23**	chrY:20842336+20842604 269bp chrY:20801568-20801824 257bp	12,11			Gerken GDB 1994; Butler et al. 2002; Kittler et al. 2003; Kayser et al. 2004, Ballantyne et al. 2010; Goedbloed et al. 2009	11	12	1

Supp. Table S3. Y-chromosome-wide dinucleotide microsatellites

NON-PALINDROMIC REPEATS	Element/ Copy	Microsatellite coordinates	Repeat unit	Repeat Number1	Element /Copy	Microsatellite coordinates	Repeat unit	Repeat Number2	Smaller allele rep. no.	Larger allele rep. no.	δr
**IR elements (IR3d: 6,102,645-6,400,947; IR3p: 9,390,341-9,757,402)**											
	IR3d2	chrY:6138991-6139024	GT	17	IR3p	chrY:9741251-9741490	GT	17	17	17	0
	IR3d3	chrY:6149872-6149905	AC	17	IR3p	chrY:9730362-9730595	AC	11	11	17	6
	IR3d4	chrY:6150010-6150048	TA	19	IR3p	chrY:9730245-9730455	TA	6	6	19	13
	IR3d5	chrY:6177482-6177528	GT	23	IR3p	chrY:9702768-9703006	GT	19	19	23	4
	IR3d6	chrY:6183213-6183252	CA	20	IR3p	chrY:9697047-9697282	CA	18	18	20	2
	IR3d7	chrY:6398638-6398684	AC	23		chrY:9468037-9468266	CA	16	16	23	7
	IR3p3	chrY:6166926-6167161	TG	11		chrY:9418244-9418277	TG	17	11	17	6
	IR3p5	chrY:6149947-6150202	AC	6		chrY:9403271-9403305	AC	17	6	17	11
**IR elements (IR4p: 8,846,281-9,171,247; IR4d: 23,729,685-24,071,881)**											
	IR4p1	chrY:23773224-23773472	CA	24	IR4 distal	chrY:9094727-9095020	CA	4	4	24	20
	IR4p3	chrY:23723445-23723699	AT	27	IR4 distal	chrY:9136577-9136792	TA	11	11	27	16
	IR4p5	chrY:24061930-24062177	AC	24	IR4 distal	chrY:9160211-9160454	AC	16	16	24	8
	IR4p6	chrY:23698802-23699047	AC	23	IR4 distal	chrY:9160211-9160454	AC	16	16	23	7
**Other Strata**											
		chrY:9697147-9697182	TG	18		chrY:6183113-6183352	TG	20	18	20	2
		chrY:9702868-9702906	AC	19		chrY:6177382-6177628	AC	23	19	23	4
		chrY:9741355-9741387	AC	16		chrY:6138894-6139120	AC	15	15	16	1
		chrY:9468135-9468169	TG	17		chrY:6398535-6398786	TG	23	17	23	6
		chrY:18296636-18296671	CA	18		chrY:18512335-18512568	CA	17	17	18	1
		chrY:18297505-18297536	AT	16		chrY:18511466-18511701	AT	20	16	20	4
		chrY:18346474-18346518	TG	22		chrY:18462481-1846272	TG	20	20	22	2
		chrY:18365542-18365582	TG	20		chrY:18443397-1844364	TG	22	20	22	2
		chrY:18368635-18368669	TG	17		chrY:18440314-1844054	TG	17	17	17	0
		chrY:23969675-23969715	AC	20		chrY:8893198-8893420	AC	4	4	20	16
		chrY:24013848-24013890	TA	21		chrY:9136577-9136792	TA	11	11	21	10
		chrY:24038489-24038536	AC	24		chrY:9160211-9160454	AC	17	17	24	7

NON-PALINDROMIC REPEATS	Element/ Copy	Microsatellite coordinates	Repeat unit	Repeat Number1	Element /Copy	Microsatellite coordinates	Repeat unit	Repeat Number2		Smaller allele rep. no.	Larger allele rep. no.	δr
		chrY:24841407-24841453	TG	23		chrY:7503752-7503992	TG	20		20	23	3
		chrY:24841407-24841453	TG	23		chrY:8832666-8832882	TG	6		6	23	17
		chrY:25647031-25647075	AC	22		chrY:7503752-7503992	AC	20		20	22	2
		chrY:28315829-28315871	TG	21		chrY:7503752-7503992	TG	20		20	21	1
**N = 28**									**Average =>**	**14.6**	**20.9**	**6.4**

PALINDROMIC REPEATS	Palindrome	Microsatellite coordinates	Repeat unit	RepeatNumber1	Element/Copy	Microsatellite coordinates	Repeat unit	RepeatNumber2		Smaller allele rep. no.	Larger allele rep. no.	δr
**Palindrome 1 (P1d:25,576,947-26,890,175; P1p:27,072,130-28,386,067)**												
P1	P1p (1)	chrY:25576947-25576983	TG	18	P1d	chrY:28385827-28386067	TG	20		18	20	2
P1	P1p (2)	chrY:25592793-25593024	TA	12	P1d	chrY:28369983-28370016	TA	17		12	17	5
P1	P1p (3)	chrY:25592862-25592901	CT	20	P1d	chrY:28369906-28370147	CT	16		16	20	4
P1	P1p (4)	chrY:25647031-25647075	AC	22	P1d	chrY:28315729-28315971	AC	21		21	22	1
P1	P1p (5)	chrY:25724455-25724492	CA	19	P1d	chrY:28237791-28238035	CA	22		19	22	3
P1	P1p (6)	chrY:25727847-25728075	TA	14	P1d	chrY:28234399-28234435	TA	18		14	18	4
P1	P1p (7)	chrY:25803119-25803151	TG	16	P1d	chrY:28159119-28159353	TG	17		16	17	1
P1	P1p (8)	chrY:25955877-25956109	AC	16	P1d	chrY:28006374-28006406	AC	16		16	16	0
P1	P1p (9)	chrY:25955977-25956009	TG	16	P1d	chrY:28006274-28006506	TG	16		16	16	0
P1	P1p (10)	chrY:25995000-25995032	TC	16	P1d	chrY:27967260-27967484	TC	14		14	16	2
P1	P1p (11)	chrY:26010269-26010310	TA	21	P1d	chrY:27951992-27952229	TA	19		19	21	2
P1	P1p (12)	chrY:26199612-26199646	TG	17	P1d	chrY:27762679-27762913	TG	17		17	17	0
P1	P1p (13)	chrY:26248368-26248398	TC	15	P1d	chrY:27713934-27714164	TC	15		15	15	0
P1	P1p (14)	chrY:26262477-26262513	TG	18	P1d	chrY:27699816-27700058	TG	21		18	21	3
P1	P1p (15)	chrY:26282901-26282941	AC	20	P1d	chrY:27679392-27679632	AC	20		20	20	0
P1	P1p (16)	chrY:26325107-26325334	AC	14	P1d	chrY:27637199-27637232	AC	17		14	17	3
P1	P1p (17)	chrY:26352381-26352426	GT	23	P1d	chrY:27609910-27610151	GT	21		21	23	2
P1	P1p (18)	chrY:26391528-26391569	AC	21	P1d	chrY:27570768-27571007	AC	20		20	21	1

PALINDROMIC REPEATS	Palindrome	Microsatellite coordinates	Repeat unit	RepeatNumber1	Element/Copy	Microsatellite coordinates	Repeat unit	RepeatNumber2		Smaller allele rep. no.	Larger allele rep. no.	δr

P1	P1p (19)	chrY:26392992-26393021	CA	15	P1d	chrY:27569316-27569545	CA	15	15	15	0
P1	P1p (20)	chrY:26421475-26421523	TG	24	P1d	chrY:27540818-27541066	TG	24	24	24	0
P1	P1p (21)	chrY:26460247-26460281	TG	17	P1d	chrY:27502057-27502291	TG	17	17	17	0
P1	P1p (22)	chrY:26499921-26499951	TG	15	P1d	chrY:27462390-27462619	TG	15	15	15	0
P1	P1p (23)	chrY:26500452-26500498	AC	23	P1d	chrY:27461837-27462089	AC	27	23	27	4
P1	P1p (24)	chrY:26538869-26538900	TA	16	P1d	chrY:27423416-27423663	TA	24	16	24	8
P1	P1p (25)	chrY:26549508-26549542	AC	17	P1d	chrY:27412766-27413000	AC	17	17	17	0
P1	P1p (26)	chrY:26594619-26594651	TG	16	P1d	chrY:27367651-27367883	TG	16	16	16	0
P1	P1p (27)	chrY:26699231-26699271	AC	20	P1d	chrY:27263023-27263265	AC	21	20	21	1
P1	P1p (28)	chrY:26890136-26890175	AT	20	P1d	chrY:27072130-27072371	AT	21	20	21	1
**Palindrome 2 (P2:25,377,686-27,193,669)**											
P2	P2p (1)	chrY:25576847-25577083	TG	18	P2d	chrY:25576947-25576983	TG	18	18	18	0
**Palindrome 3 (P3:23,977,880-24,873,141)**											
P3	P3p (1)	chrY:24115165-24115210	CA	23	P3d	chrY:24763378-24763615	CA	19	19	23	4
P3	P3p (2)	chrY:24177698-24177732	TG	17	P3d	chrY:24700857-24701091	TG	17	17	17	0
P3	P3p (3)	chrY:24193829-24193859	TG	15	P3d	chrY:24684729-24684959	TG	15	15	15	0
P3	P3p (4)	chrY:24205483-24205512	TG	15	P3d	chrY:24673074-24673305	TG	16	15	16	1
P3	P3p (5)	chrY:24267224-24267253	CA	15	P3d	chrY:24611357-24611586	CA	15	15	15	0
P3	P3p (6)	chrY:24326955-24326992	CA	19	P3d	chrY:24551619-24551858	CA	19	19	19	0
**Palindrome 4 (P4:20,611,929-21,032,220)**											
P4	P4p (1)	chrY:20631938-20631980	AC	21	P4d	chrY:21012191-21012231	AC	20	20	21	1
P4	P4p (2)	chrY:20637579-20637629	AT	25	P4d	chrY:21006542-21006592	AT	25	25	25	0
P4	P4p (3)	chrY:20637630-20637661	GT	16	P4d	chrY:21006510-21006541	GT	16	16	16	0
P4	P4p (4)	chrY:20654982-20655023	AT	21	P4d	chrY:20989155-20989192	AT	19	19	21	2
P4	P4p (5)	chrY:20655023-20655069	TG	23	P4d	chrY:20989105-20989155	GT	25	23	25	2
P4	P4p (6)	chrY:20712288-20712320	AC	16	P4d	chrY:20931854-20931890	AC	18	16	18	2
P4	P4p (7)	chrY:20778782-20778817	TG	18	P4d	chrY:20865355-20865390	TG	18	18	18	0

PALINDROMIC REPEATS	Palindrome	Microsatellite coordinates	Repeat unit	RepeatNumber1	Element/Copy	Microsatellite coordinates	Repeat unit	RepeatNumber2	Smaller allele rep. no.	Larger allele rep. no.	δr
**Palindrome 5 (P5d: 19,567,357-20,066,326;P5p: 20,063,027-20,612,242)**											
P5	P5p (1)	chrY:19622111-19622156	CA	23	P5d	chrY:20557622-20557659	TG	19	19	23	4

P5	P5p (2)	chrY:19950183-1,950219	TG	18	P5d	chrY:20229558-20229594	AC	18		18	18	0
P5	IR5p (1)	chrY:19997563-19997597	TG	17	P5d	chrY:20132166-20132204	AC	19		17	19	2
P5	IR5p (2)	chrY:20021497-20021527	TA	15	P5d	chrY:20108240-20108270	AT	15		15	15	0
**Palindrome 6 (P6:18,271,274-18,537,845)**												
P6	P6p (1)	chrY:18296636-18296671	CA	18	P6d	chrY:18512435-18512468	TG	17		17	18	1
P6	P6p (2)	chrY:18297505-18297536	AT	16	P6d	chrY:18511466-18511701	TA	19		16	19	3
P6	P6p (3)	chrY:18346474-18346518	TG	22	P6d	chrY:18443497-18443541	AC	22		22	22	0
P6	P6p (4)	chrY:18365542-18365582	TG	20	P6d	chrY:18440414-18440448	AC	17		17	20	3
**Palindrome 8 (P8:16,095,787-16,170,613)**												
P8	P8p (1)	chrY:16099088-16099134	TG	23	P8d	chrY:16167356-16167402	AC	23		23	23	0
**N = 51**									**Average =>**	**17.8**	**19.2**	**1.4**

STATISTICS

	∂r=0	∂r≠0	Total
Non-palindromic repeats	2	26	28
Palindromic repeats	23	28	51
**Fisher Exact test: odds ratio 0.0961:**	**P=0.00038 (p<0.001***)**		

Supp. Table S1. Y-chromosomal haplotype data is available as an Excel file under the Supporting Information for this article.
